# III–V-on-Silicon Photonic Integrated Circuits for Spectroscopic Sensing in the 2–4 μm Wavelength Range

**DOI:** 10.3390/s17081788

**Published:** 2017-08-04

**Authors:** Ruijun Wang, Anton Vasiliev, Muhammad Muneeb, Aditya Malik, Stephan Sprengel, Gerhard Boehm, Markus-Christian Amann, Ieva Šimonytė, Augustinas Vizbaras, Kristijonas Vizbaras, Roel Baets, Gunther Roelkens

**Affiliations:** 1 Photonics Research Group, Ghent University-imec, Technologiepark-Zwijnaarde 15, Ghent 9052, Belgium; Ruijun.Wang@ugent.be (R.W.); Anton.Vasiliev@ugent.be (A.V.); Muhammad.Muneeb@ugent.be (M.M.); Amalik@ece.ucsb.edu (A.M.); Roel.Baets@UGent.be (R.B.); 2 Center for Nano- and Biophotonics (NB-Photonics), Ghent University, Ghent 9000, Belgium; 3 Walter Schottky Institut, Technische Universität München, Am Coulombwall 4, Garching 85748, Germany; Stephan.Sprengel@wsi.tum.de (S.S.); Gerhard.Boehm@wsi.tum.de (G.B.); Amann@wsi.tum.de (M.-C.A.); 4 Brolis Semiconductors UAB, Moletu pl. 73, Vilnius LT-14259, Lithuania; ieva.simonyte@brolis-semicon.com (I.Š.); augustinas.vizbaras@brolis-semicon.com (A.V.); kristijonas.vizbaras@brolis-semicon.com (K.V.)

**Keywords:** silicon photonics, mid-infrared, optical sensor, integrated spectrometer, spectroscopy

## Abstract

The availability of silicon photonic integrated circuits (ICs) in the 2–4 μm wavelength range enables miniature optical sensors for trace gas and bio-molecule detection. In this paper, we review our recent work on III–V-on-silicon waveguide circuits for spectroscopic sensing in this wavelength range. We first present results on the heterogeneous integration of 2.3 μm wavelength III–V laser sources and photodetectors on silicon photonic ICs for fully integrated optical sensors. Then a compact 2 μm wavelength widely tunable external cavity laser using a silicon photonic IC for the wavelength selective feedback is shown. High-performance silicon arrayed waveguide grating spectrometers are also presented. Further we show an on-chip photothermal transducer using a suspended silicon-on-insulator microring resonator used for mid-infrared photothermal spectroscopy.

## 1. Introduction

Silicon photonics has attracted great interest as a promising integrated-optics platform for various applications in the telecommunication wavelength range, such as optical interconnects [1,2]. This platform can take advantage of the silicon electronics processes to fabricate photonic devices in high yield and high volume. In addition, the high refractive index contrast of silicon-on-insulator (SOI) waveguides enables a tight bending radius, and consequently ultra-compact photonic devices and systems. With these advantages, in recent years the potential applications of silicon photonic integrated circuits (ICs) are extended to areas such as gas sensing [3,4,5], bio-sensing [6,7] and biomedical diagnostics [8,9]. However, the silicon photonic devices used at telecommunication wavelengths are typically retained for most of these new applications due to the lack of components at other wavelengths, which limits the performance of these photonic systems. For example, a silicon photonic on-chip sensor for evanescent field absorption spectroscopy of CH_4_ near 1.65 μm was recently demonstrated [10]. A sensitivity of 756 ppmv∙Hz^−1/2^ is obtained by using a 10 cm long silicon spiral waveguide. Extending the operation wavelength of the silicon photonic on-chip sensor to around 2.35 μm or 3.25 μm would enable a more compact on-chip sensor with higher sensitivity since the absorption coefficient of CH_4_ at these longer wavelengths is much higher than that at 1.65 μm [11]. The mid-infrared spectral range contains strong absorption features of many gases and chemicals that are of great interest for industrial, medical and environmental applications [12,13,14]. The realization of silicon photonics components in this wavelength range could enable economical and compact optical sensors with superior performance in sensitivity, power consumption and portability compared to current bulky solutions [15,16,17]. In standard absorption spectroscopy, the noise of the photodetector often limits the sensitivity of the system and often cooled detectors are required to meet the system requirements. In view of further miniaturization and cost reduction, novel indirect methods to measure the optical absorption such as photoacoustic and photothermal spectroscopy are gaining popularity. These methods are shown to be highly sensitive and robust to environmental noise for the detection of various chemical compounds [18,19,20,21,22,23,24,25]. Part-per-trillion (ppt) trace gas sensitivities have been demonstrated using a quartz-enhanced photoacoustic spectroscopy (QEPAS) system [25].

The SOI waveguide circuit platform has become a standard for integrated photonics in the telecommunication wavelength range and the processes to fabricate these devices inside silicon photonics foundries are well developed [26]. The development of mid-infrared (>2 μm wavelength) optical sensors based on SOI waveguide circuits can benefit from these well-established processes. But the absorption of silicon dioxide rapidly increases and substrate leakage loss also becomes an issue at wavelengths beyond 4 μm [27]. Specific structures should be used to reduce the SOI waveguide loss [28,29,30,31,32,33]. Also, importantly, light sources and photodetectors need to be integrated on the SOI platform. This typically involves the integration of III–V semiconductors on the silicon photonic platform. In this paper we summarize our recent work on 2–4 μm wavelength range III–V-on-silicon photonic integrated circuits based on the SOI platform. The paper is organized as follows. Section 2 introduces fully integrated mid-infrared photonic circuits for chip-scale optical sensors. The third section focuses on the heterogeneously integrated 2.3 μm III–V laser sources and photodetectors on silicon waveguide circuits. The fourth section presents a compact 2 μm wavelength widely tunable external cavity laser using a silicon photonic IC for the wavelength selective feedback. Then silicon arrayed waveguide grating (AWG) spectrometers operating in the 2–4 μm wavelength range are presented in the fifth section, together with the integration of InP-based and GaSb-based photodetector arrays. In the last section, a photothermal mid-infrared spectroscopy method is presented that uses a suspended SOI microring resonator operating at 1.55 μm acting as a transducer for photothermal spectroscopy in the 3–4 μm wavelength range, circumventing the need for a cooled mid-infrared detector.

## 2. Mid-Infrared Silicon Photonic Integrated Circuits

**Figure 1:**
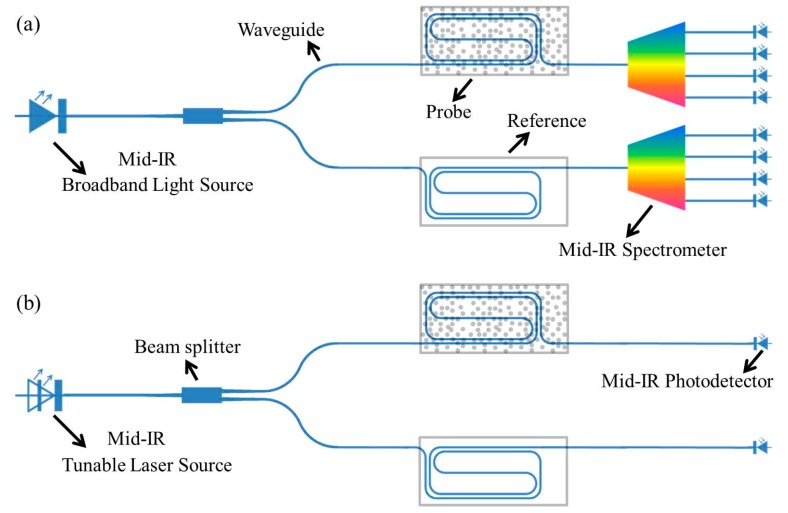
(**a**) Schematic of two silicon photonic configurations to realize an integrated on-chip mid-infrared absorption spectroscopy sensor. Broadband source and spectrometer, best suited for liquid and solid analytes; (**b**) Tunable single mode laser source for trace gas detection.

In most applications, a spectroscopic sensing system should have a light source, a probe component and a spectrometer or single pixel detector. In previously demonstrated on-chip optical sensors, light from an external light source is coupled to the probe component (waveguide circuit), which interacts with the environment, and afterwards is read by an external detector. For a compact sensor system, both the light source and detector should be integrated together with the waveguide circuit. Figure 1 shows two typical configurations of fully integrated mid-infrared on-chip spectroscopic sensors. In both configurations, light is coupled from the integrated light source to the waveguide and then split to two arms. The probe component in one arm interacts with environment while the other one provides the reference information. Different probe components have been proposed to realize efficient interaction between the environment and light in the waveguide, e.g., slot waveguides [34,35], spiral waveguides [10,36], and microring resonators (MRRs) [4,5,37]. In the case of liquid sensing, a low-cost broadband light source such as a light emitting diode (LED) can be used since liquid samples typically have broad absorption features. In this configuration, a spectrometer with integrated photodetectors should be implemented to analyze the absorption spectra. For gas sensing, typically a tunable single mode laser is required to probe the absorption lines of gases, as used in the popular tunable diode laser absorption spectroscopy (TDLAS) technique. Integrating a widely tunable laser or a broadband wavelength coverage laser array on the waveguide circuit enables to simultaneously detect several gases or even broad absorption features of liquids using the configuration shown in Figure 1b.

For mid-infrared silicon photonic ICs, low-loss passive waveguides, beam splitters and filters (spectrometers) can be fabricated in silicon foundries [17], which is an asset of mid-infrared silicon photonic sensors. At 2 μm wavelength, a propagation loss of 0.6 dB/cm was achieved for SOI strip waveguides (TE-polarization) [38]. As the wavelength increases to 3.8 μm, a low prorogation loss of ~1.5 dB/cm for 400 nm rib SOI waveguides with 220 nm etch depth was reported by Nedeljkovic et al. [39].

## 3. III–V-on-Silicon Platform for the 2 μm Wavelength Range

**Figure 2:**
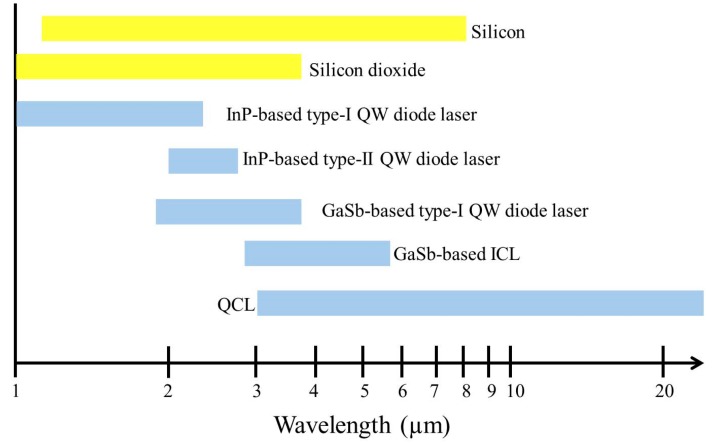
Transparent window of silicon and silicon dioxide, and emission wavelength coverage of semiconductor lasers based on different III–V active regions. InP-based type-I, type-II and GaSb-based type-I quantum well (QW) diode lasers, GaSb-based interband cascade lasers (ICLs), and QCLs are included.

Silicon is an indirect bandgap semiconductor with extreme low light emission efficiency, transparent beyond 1.1 μm wavelength. In order to realize fully integrated silicon photonic systems, a few approaches have been developed to integrate active opto-electronic devices on silicon, especially in the telecommunication wavelength range. This includes the direct epitaxial growth of III–V or Germanium-based material [40,41,42], the heterogeneous integration of III–V material on silicon [43,44] and the flip-chip integration of prefabricated semiconductor devices [45]. Among these approaches, the heterogeneous integration of III–V material on silicon through bonding has been proven to be a promising solution to integrate opto-electronic devices on silicon photonic ICs. In this way, heterogeneously integrated III–V-on-silicon lasers and photodetectors can be realized using the same epitaxial layer stack. In the mid-infrared wavelength range, quantum cascade structures and interband cascade structures can be used as the active region for high-performance lasers above 3 μm wavelength [46,47], while InP-based type-I, type-II and GaSb-based type-I heterostructures can provide the gain for diode lasers in the 2–3 μm wavelength range [48,49,50], as shown in Figure 2. Recently, A. Spott et al. reported a quantum cascade laser (QCL) heterogeneously integrated on a silicon-on-nitride-on-insulator waveguide circuit, with emission wavelength between 4.6 and 4.9 μm [51]. 

In this paper, we focus for the laser integration on the 2–2.5 μm wavelength range which is relevant for many gas sensing applications (including for example CO_2_, CO, HF and NH_3_). In this wavelength range, GaSb-based type-I diode lasers exhibit high performance. However, the heterogeneous integration of GaSb-based laser material on silicon is far from mature, which results in low process yield and non-ideal performance. The InP material system is far better understood, especially in the context of heterogeneous integration. A heterogeneously integrated III–V-on-silicon laser and amplifier near 2 μm using InP-based strained InGaAs type-I heterostructures was reported [52,53]. However, the emission wavelength of highly strained quantum wells grown on InP substrate is limited to around 2.3 μm [54]. In recent years, type-II quantum well lasers grown on InP substrate with emission wavelength up to 2.7 μm were demonstrated [55]. Besides, resonant-cavity light emitting diodes operating up to 3.3 μm wavelength and photoluminescence up to 3.9 μm wavelength were reported based on these InP-based type-II InGaAs/GaAsSb quantum wells [49,56]. These results indicate that InP-based type-II heterostructures are promising for the integration of 2–4 μm wavelength range light sources on a silicon photonic integrated circuit. 

**Figure 3:**
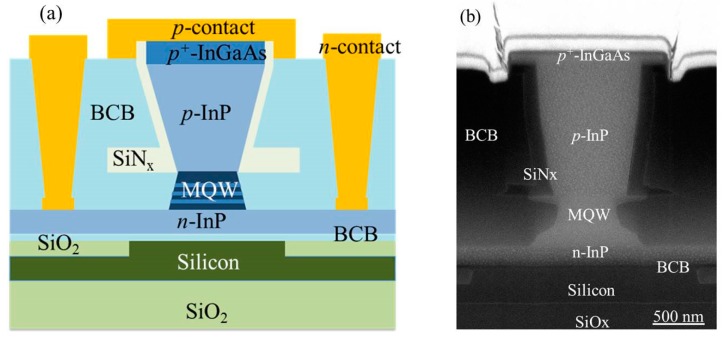
(**a**) Schematic drawing and (**b**) scanning electron microscopy (SEM) image of the cross section of a heterogeneously integrated III–V-on-silicon type-II active device.

Recently, we demonstrated 2.3 μm InP-based type-II laser sources and photodetectors heterogeneously integrated on silicon photonic ICs [57,58,59]. Figure 3a shows the schematic cross-section of the heterogeneous InP-based type-II device on a SOI waveguide. The III–V epitaxial layer stack consists of a 200 nm thick *n*-InP contact layer, a type-II active region surrounding by two separate confinement heterostructures, a 1.5 μm thick *p*-InP cladding layer and a 100 nm *p*-InGaAs contact layer. The active region contains six pairs of “W”-shaped InGaAs/GaAsSb quantum wells. The silicon waveguide circuit is fabricated in IMEC’s CMOS pilot line on a 200 mm SOI wafer, with a 180 nm deep dry etch in the 400 nm thick silicon device layer. The III–V epitaxial layer stack is adhesively bonded to a SOI waveguide using an ultra-thin divinylsiloxane-benzocyclobutene (DVS-BCB) layer of a few tens of nanometers. After bonding, the InP-substrate is removed by HCl wet etching. Then the lasers and photodetectors are co-processed in the III–V membrane. First, an anisotropic HCl wet etch of the *p*-InP cladding layer is employed to create a “V”-shaped mesa. Then the active region is etched by using a 1:1:20:70 H_3_PO_4_:H_2_O_2_:citric acid:H_2_O solution. Subsequently, the devices are isolated by wet etching of the *n*-InP layer using HCl. A combination of SiNx and DVS-BCB is used to passivate the device mesa. A SEM image of the cross-section of the fabricated III–V-on-silicon devices is shown in Figure 3b.

### 3.1. Heterogeneously Integrated 2.3 μm Range Distributed Feedback Lasers and Laser Arrays

**Figure 4:**
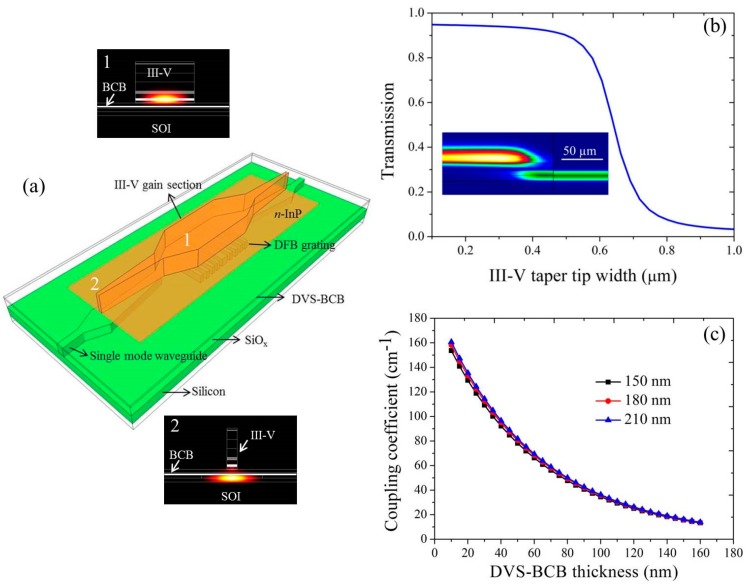
(**a**) Schematic of an InP-based type-II DFB laser heterogeneously integrated on a silicon waveguide, the simulated mode intensity distribution in different sections is also included; (**b**) simulated coupling efficiency of a 180 μm long III–V/silicon SSC as a function of the III–V taper tip width. The inset figure shows the fundamental mode intensity evolution of the SSC with 0.5 μm wide III–V taper tip; (**c**) calculated coupling strength of the DFB grating as a function of the DVS-BCB thickness for three different etch depths (150, 180, 210 nm) in the 400 nm silicon device layer.

Distributed feedback (DFB) lasers are well-suited light sources for the TDLAS measurement of gases [60]. Figure 4a shows the schematic of an InP-based type-II DFB laser heterogeneously integrated on a silicon waveguide [59]. The device can be divided into two parts: a gain section at the center and III–V/silicon spot size converters (SSCs) on both sides. In the gain section, most of the optical mode is confined in the III–V waveguide. A quarter-wave shifted first-order DFB grating with an etch depth of 180 nm in a 400 nm silicon device layer are implemented beneath the gain section. The evanescent tail of the optical mode interacts with this grating, which selects the lasing wavelength of the DFB laser. An efficient light coupling from the III–V waveguide to the silicon waveguide is realized using the III–V/silicon SSCs by tapering both waveguides. The III–V/silicon SSCs have two sections [58]. In the first section, the III–V waveguide is linearly tapered from 5 μm to 1.2 μm over a length of 50 μm. In the second section, the III–V waveguide is slowly tapered to a narrow tip while the silicon waveguide is tapered from 0.2 μm to 3 μm over a length of 180 μm. Figure 4b shows the simulated transmission efficiency of the III–V/silicon SSC as a function of the width of III–V taper tip. It can be found that the coupling efficiency is higher than 90% when the width of III–V taper tip is narrower than 0.5 μm. A longitudinal cross section of the III–V/silicon SSC with a 0.5μm wide tip is shown in the inset of Figure 4b, indicating the evolution of the fundamental mode. For DFB lasers, the threshold current and output power depends on the coupling coefficient of the grating [61]. The calculated coupling coefficient as a function of the DVS-BCB thickness and grating etch depth is shown in Figure 4c. With a 50 nm thick bonding interface, the coupling coefficient of the DFB grating is around 78 cm^−1^, which reduces to 35 cm^−1^ as the thickness of DVS-BCB thickness increases to 100 nm.

**Figure 5:**
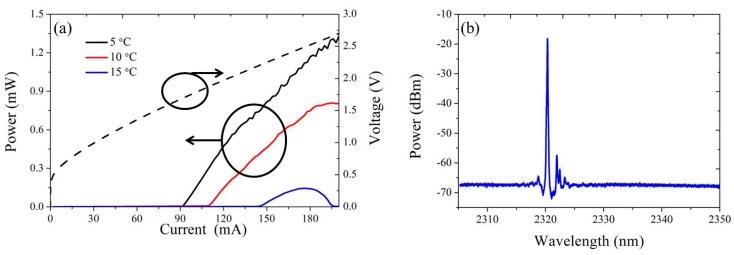
(**a**) Continuous-wave (CW) light-current-voltage (L-I-V) curve of a III–V-on-silicon DFB laser with grating period of 348 nm; (**b**) emission spectrum of the DFB laser driven with 190 mA bias current at 10 °C.

Figure 5a shows the CW L-I-V curve of a heterogeneously integrated InP-based type-II DFB laser with a 1000 μm long gain section, 5 μm wide III–V mesa, 60 nm thick DVS-BCB layer and grating pitch of 348 nm. At 5 °C, the threshold current of the DFB laser is 90 mA, which corresponds to a current density of 1.8 kA/cm^2^. The maximum on-chip output power is around 1.3 mW. A high resolution emission spectrum of the DFB laser biased at 190 mA at a temperature of 10 °C is shown in Figure 5b. Single mode lasing with a side mode suppression ration (SMSR) of 40 dB is achieved. From the emission spectrum, a normalized coupling coefficient *κL* of 5.5 can be deduced, which is quite close to the calculated value (6.2) based on the data shown in Figure 4c. The maximum CW operating temperature of the DFB laser is around 17 °C. For DFB lasers, the threshold current density will reduce and the maximum CW operating temperature will become higher when the *κL* increases [61]. In recently fabricated DFB lasers with a 50 nm DVS-BCB bonding layer, the maximum CW operating temperature increases to 25 °C while the threshold current density reduces to 1.6 kA/cm^2^ at 5 °C [62]. However, higher *κL* introduces spatial-hole burning in the DFB laser. The lasing wavelength can hop from the defect mode to the band edge mode as the bias current increases. A maximum single mode output power of ~3 mW is achieved in the band edge mode [62].

**Figure 6:**
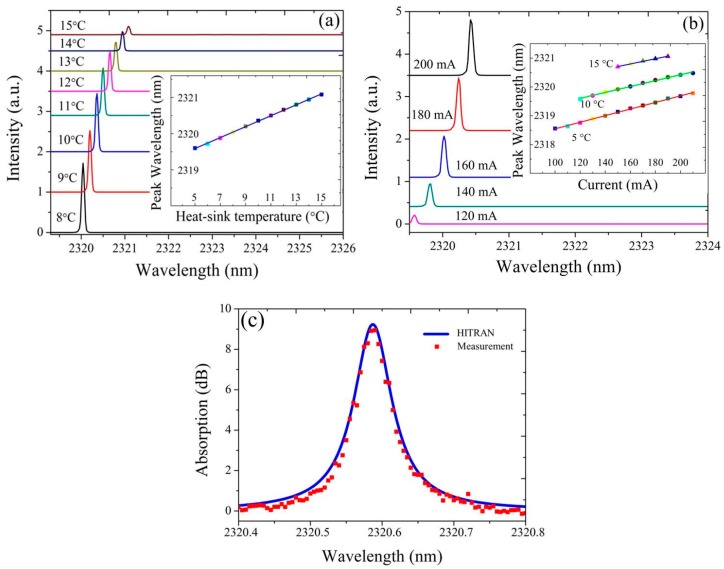
(**a**) Evolution of the DFB laser emission spectrum with increasing heat-sink temperature under 190 mA bias current, (**b**) with increasing bias current at 5 °C. The inset figure shows the dependence of the lasing wavelength on (**a**) temperature and (**b**) bias current at 5 °C, 10 °C, 15 °C; (**c**) direct TDLAS measurement of CO and the corresponding HITRAN spectrum.

In Figure 6a,b, the emission spectrum of a DFB laser with a grating period of 348 nm and 60 nm bonding layer is plotted for a range of heat-sink temperatures and bias currents. The dependence of the lasing wavelength on temperature is shown in the inset of Figure 6a. A temperature-tuning rate of 0.15 nm/°C is fitted. In the inset of Figure 6b, the laser emission wavelength as a function of the bias current at a heat-sink temperature of 5 °C, 10 °C and 15 °C is shown. The measured current-tuning rate is about 0.01 nm/mA. As the temperature increases from 5 °C to 15 °C, single mode lasing is observed over the whole bias current range. With this stable tuning behavior, the heterogeneously integrated DFB laser can be used as the light source for TDLAS measurement of gases. We carried out the first gas sensing measurement using such a silicon photonic laser source. In the measurement, the light from the DFB laser is coupled to a single mode fiber and then coupled into and out of a gas cell through a collimator. The 10 cm long gas cell contains pure CO at 1 atm and has wedged AR coated windows. The heat-sink temperature of the DFB laser is fixed at 13 °C. Using the measured dependencies of lasing wavelength on heat-sink temperature and bias current, the TDLAS spectrum of CO is recovered in Figure 6c. It can be found that the measurement result fits the data from the HITRAN database very well.

**Figure 7:**
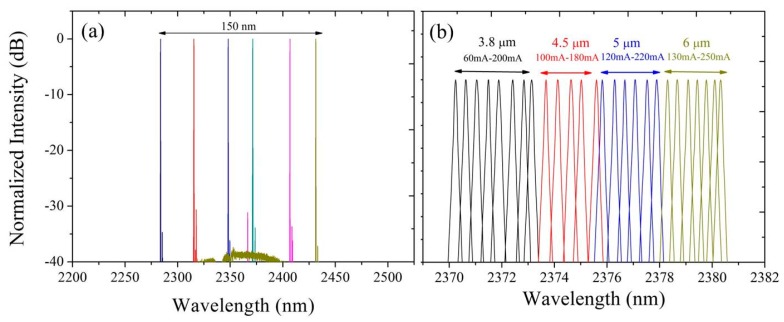
(**a**) Emission spectra of six 1000 μm-long DFB lasers with different silicon grating period in an array; (**b**) evolution of the emission spectrum with bias current for four 700 μm long DFB lasers with different III–V waveguide widths in an array.

The limited tuning range of a DFB laser limits the number of gases that can be detected with the source. The development of broadly tunable laser sources enables multi-species trace gas detection and bio-sensing. Recently, we demonstrated broad wavelength coverage III–V-on-silicon DFB laser arrays [62]. Figure 7a shows the emission spectra of six 1000 μm-long heterogeneously integrated DFB lasers in an array. All lasers are driven in CW, biased at 150 mA and operated at 5 °C. As the silicon grating pitch increases from 343 nm to 368 nm, the lasing wavelength shifts from 2280 nm to 2430 nm. This broad wavelength span overlaps with absorption features of several important gases, such as NH_3_, CO, CH_4_, C_2_H_2_ and HF. As seen in Figure 7a, a 1 nm change in the silicon grating pitch results in a 6 nm shift in the emission wavelength. In addition, the tuning range of a single DFB laser is around 3 nm. Therefore, a 0.5 nm increment in the silicon grating pitch is required to get a continuously tunable DFB array, which is quite challenging for current silicon photonic pilot lines. Besides adjusting the grating pitch, another method to control the lasing wavelength is to adjust the gain section width of the DFB lasers. Figure 7b shows the CW lasing spectra of four 700 μm-long DFB lasers with different III–V waveguide widths as a function of bias current. More than 10 nm continuous current-tuning range is achieved this way. 

### 3.2. III–V-on-Silicon Photodetectors in the 2 μm Wavelength Range

**Figure 8:**
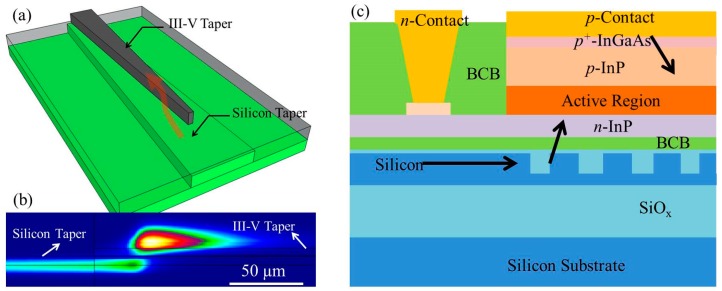
(**a**) Schematic of an adiabatically-coupled photodetector integrated on a silicon waveguide; (**b**) mode intensity distribution in a longitudinal cross section of the III–V/silicon taper taking the active region absorption into account; (**c**) schematic cross-section of a grating-assisted III–V-on-silicon photodetector.

Besides light sources, photodetectors are another type of opto-electronic devices that should be integrated on photonic ICs for compact silicon photonic spectroscopic sensors, as discussed in Section 2. We use the same epitaxial layer stack as the heterogeneously integrated InP-based type-II DFB laser to realize 2 μm wavelength range III–V-on-silicon photodetectors [55,63]. This way, all of the opto-electronic components required for a spectroscopic sensor can be realized using a single epitaxial layer stack and process flow. Two different coupling methods between the silicon waveguide and the III–V active region are compared in our study [63]. The first structure is based on an adiabatic taper as shown in Figure 8a. The III–V mesa of the photodetector is a 150 μm long waveguide, tapered from 1 μm to 3.5 μm. In this device, light is efficiently coupled from the silicon waveguide into the III–V waveguide by a narrow taper tip, and subsequently absorbed by the active region. Figure 8b shows a simulated intensity distribution of the optical field along the adiabatic taper when the absorption of the active region is taken into account. The simulation result indicates that more than 95% of the light is absorbed in the III–V active region. The other III–V-on-silicon photodetector structure is based on a grating-assisted coupling as shown in Figure 8c. In this structure, light in the silicon waveguide is diffracted to the III–V active region by using a silicon grating. The size of the diffraction grating and photodetector mesa is 20 × 20 μm and 25 × 25 μm, respectively. Compared to the adiabatic-taper-based photodetector, the grating-assisted photodetector has better fabrication tolerance since it does not require a narrow III–V taper tip and very fine alignment between the III–V and silicon waveguide.

**Figure 9:**
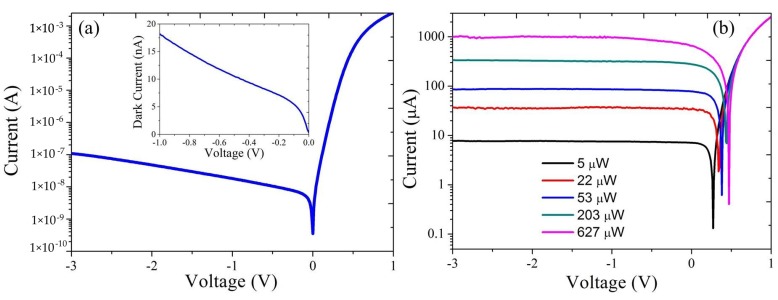
(**a**) I-V curve of the heterogeneously integrated adiabatic-taper-based photodetector in the dark, the inset shows the dark current of the device from −1 V to 0 V; (**b**) I-V curve of the photodetector under different waveguide-coupled input powers at a wavelength of 2.35 μm.

A typical I-V characteristic of the adiabatic-taper-based photodetector in the dark at room temperature is shown in Figure 9a. The dark current is 10 nA at a reverse voltage of −0.5 V and increases to 100 nA at −3 V. Figure 9b shows the I-V curve of the photodetector under different waveguide-referred input power levels at 2.35 μm wavelength. The waveguide-referred responsivity is around 1.6 A/W. At 0 V bias, the photodetector response is linear to the input optical power up to 200 μW. As the input power increases, higher reverse voltage is required to extract all photogenerated carriers. Under a reverse bias voltage of 1 V, the photodetector has linear response up to an input power of around 630 μW, which is the maximum optical power that can be obtained from our laser source.

The grating-assisted photodetector has a dark current of 5 nA under −0.5 V bias and responsivity of 0.1 A/W at 2.35 μm wavelength. The lower dark current compared to that of the adiabatically-coupled photodetector can be ascribed to the smaller perimeter. The responsivity of the grating-assisted photodetector is determined by the out-coupling efficiency of the diffraction grating (simulated to be 40%) towards the detector and the limited thickness of the absorbing active region layer. The active region of the epitaxial layer stack used for the III–V-on-silicon photodetectors is a ~100 nm thick type-II quantum well layer stack, thereby resulting in a low responsivity. Increasing the thickness of the absorbing III–V layer and introducing a bottom mirror under the diffraction grating can be used to improve the quantum efficiency of the grating-assisted photodetector [64].

## 4. GaSb/Silicon Hybrid External Cavity Laser

In order to achieve a wide wavelength tuning range, mid-infrared semiconductor lasers are typically assembled in two configurations: a DFB laser array and an external cavity laser structure [46]. In the previous section, we presented our work on broad wavelength coverage with a 2.3 μm III–V-on-silicon DFB laser array. In this section, we will introduce our recent results on a 2 μm GaSb/silicon hybrid external cavity laser [65]. In this work, a silicon photonic IC is used as the external cavity to replace traditional bulky configurations (such as the Littrow configuration) and their mechanical controllers [66,67], thereby leading to a miniaturized and high-performance widely tunable external cavity laser.

**Figure 10:**
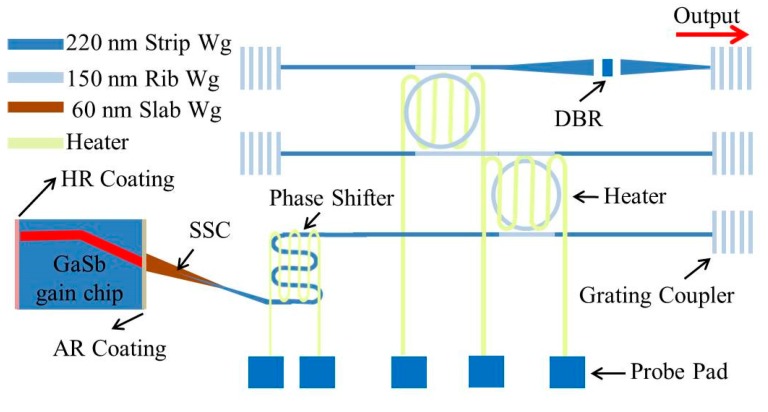
Schematic of a GaSb/silicon hybrid external cavity laser.

The external cavity laser consists of a GaSb-based gain chip and a silicon photonic IC for the wavelength-selective feedback, as schematically shown in Figure 10. In the gain chip, a HR coating with >95% reflectivity is applied on one facet while an AR coating with <0.1% reflectivity is applied on the other facet close to the silicon photonic IC. The III–V waveguide is tilted 5.2 degrees at the AR-coated facet. The light coupling between the gain chip and silicon photonic IC is realized by a silicon SSC. At the silicon SSC facet, a 6 μm × 0.06 μm slab waveguide tilted 12 degrees is used to achieve efficient butt-coupling and reduce back-reflections. Then a 200 μm long silicon waveguide tapered from 180 nm to 700 nm is used to convert the mode from the slab waveguide to the single mode of the strip waveguide. Simulations indicate that the butt-coupling loss at the GaSb/silicon slab waveguide interface is 1 dB while the mode conversion loss in the slab/strip waveguide is also around 1 dB. The silicon photonic IC contains a Vernier filter consisting of two thermally tuned micro-ring resonators (MRRs), a phase section to allow for quasi-continuous wavelength tuning and a silicon distributed Bragg reflector (DBR). A Fabry-Perot laser cavity is formed between the HR-coated facet of the gain chip and the silicon waveguide DBR. Ti/Au micro-heaters are integrated on the Vernier filter to thermally control the overlapping wavelength of the transmission spectra of the two MRRs, thereby leading to broad tuning of the lasing wavelength. In our device, the free spectral range (FSR) of the two MRRs is 6 nm and 6.5 nm, respectively.

**Figure 11:**
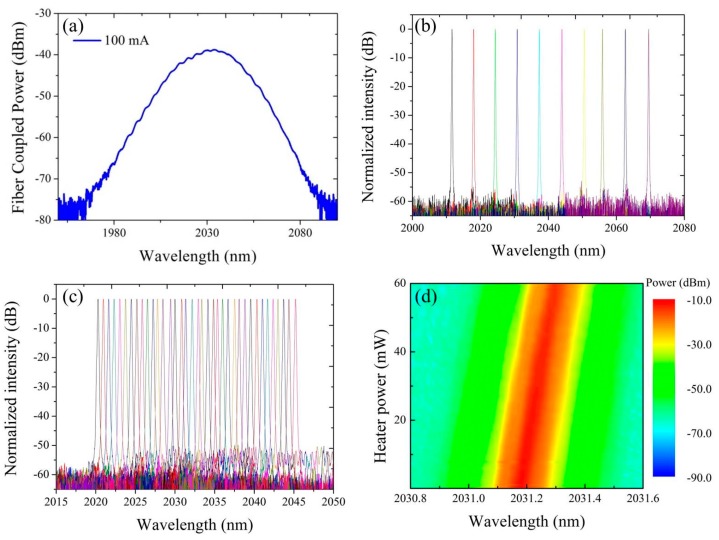
(**a**) Amplified spontaneous emission coupled from the GaSb-based SLD to a silicon waveguide; (**b**) superimposed spectra of the hybrid laser by thermally tuning only one MRR; (**c**) both MRRs and (**d**) phase shifter.

Figure 11a shows the amplified spontaneous emission spectrum coupled from the GaSb-based gain chip to a silicon waveguide (without the DBR and Vernier filter) through the silicon SSC. It can be found that the spectrum is smooth without any sign of lasing modes, indicating low parasitic reflections at the GaSb/silicon interface.

The broadband gain can e.g., cover a large part of the absorption window of CO_2_ in the 2 μm wavelength range. By implementing the waveguide-based DBR and Vernier filter in the silicon photonic feedback circuit, a tunable single mode laser can be obtained. Figure 11b shows the superimposed lasing spectra of the GaSb/silicon hybrid laser by tuning one MRR. A tuning range of 58 nm is achieved with a SMSR better than 52 dB over the full tuning range and more than 60 dB at the optimal wavelength. When only one MRR is tuned, the tuning resolution is determined by the FSR of the other MRR. Fine quasi-continuous wavelength tuning can be achieved by simultaneously tuning both MRRs. Figure 11c shows the superimposed spectra with 0.7 nm resolution tuning over 25 nm range realized in this way. But even when tuning both MRRs, the tuning resolution is still limited by the FSR of the longitudinal modes of the Fabry-Perot cavity. Continuous tuning can be achieved by thermally tuning the phase section to continuously adjust the Fabry-Perot cavity length. A spectral map of the fiber-coupled laser emission as a function of the dissipated power on the phase section is shown in Figure 11d illustrating the continuous tuning. All results shown in Figure 11b–d are achieved from an external cavity laser with a coupling gap of 500 nm between the MRRs and bus waveguides. This laser has a maximum output power of 3.8 mW and threshold current density of 1 kA/cm^2^ in uncooled condition. When the coupling gap of the MRRs is reduced to 200 nm, the output power increases to 7.5 mW while the threshold current density reduces to 0.8 kA/cm^2^. But the SMSR and tuning smoothness are lowered as the coupling gap is reduced.

## 5. Mid-Infrared AWG Spectrometers in the 2–4 μm Wavelength Range

For spectroscopy applications involving broad absorption features such as liquids and solids, the most cost-efficient solution includes a broadband light source and a suitable spectrometer to analyze the spectral response as shown in Figure 1a. As an example, the typical 3 dB bandwidth of absorption features of a liquid such as sesame oil at 3.4 μm and an organic solid such as PDMS in the 2.3 μm region would be about 900 GHz (35 nm) and 1300 GHz (50 nm), respectively [68]. A spectrometer resolution of 200 GHz would be adequate in both cases. In recent years, considerable effort has been devoted to develop novel spectrometers that are compact and can be integrated with photonic ICs [17,69,70]. It is possible to realize different types of spectrometers in the SOI platform for the 2–4 μm wavelength range such as spatial heterodyne spectrometers (SHS), planar concave gratings (PCGs) and AWGs [71,72,73]. AWGs are widely used as (de)multiplexers in wavelength division multiplexing (WDM) systems and can also be used as spectrometers in spectroscopic sensors [74]. In addition, AWGs are also used as multiplexers for DFB laser arrays. Combining a mid-infrared DFB laser array with an AWG allows coupling the light from different lasers to a single diffraction limited output waveguide with low loss [75].

The AWG consists of two free propagation regions (FPRs), connected together through an array of delay waveguides with constant length increment between them. When the light enters the input FPR through an input channel, it diverges and couples into the array of delay waveguides. The constant length increment between delay waveguides introduces a constant change of phase between the arms, which depends on the wavelength. As a result, light diffracted from each delay waveguide interferes constructively and gets refocused in the output FPR at different output waveguides. Detailed information about the AWG design can be found in [76].

**Figure 12:**
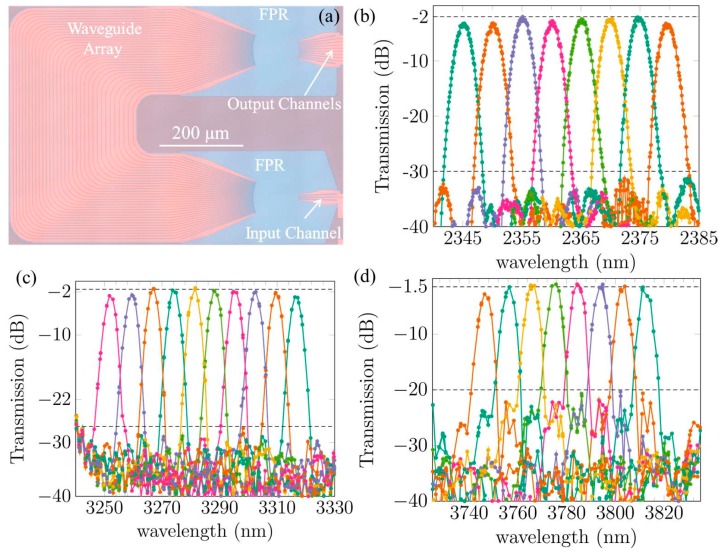
(**a**) Microscope image of a 2.3 μm silicon arrayed waveguide grating (AWG) spectrometer; the measured spectral responses of all the channels in three AWGs operating at different wavelengths: (**b**) 2.3 μm; (**c**) 3.3 μm and (**d**) 3.8 μm.

**Figure 13:**
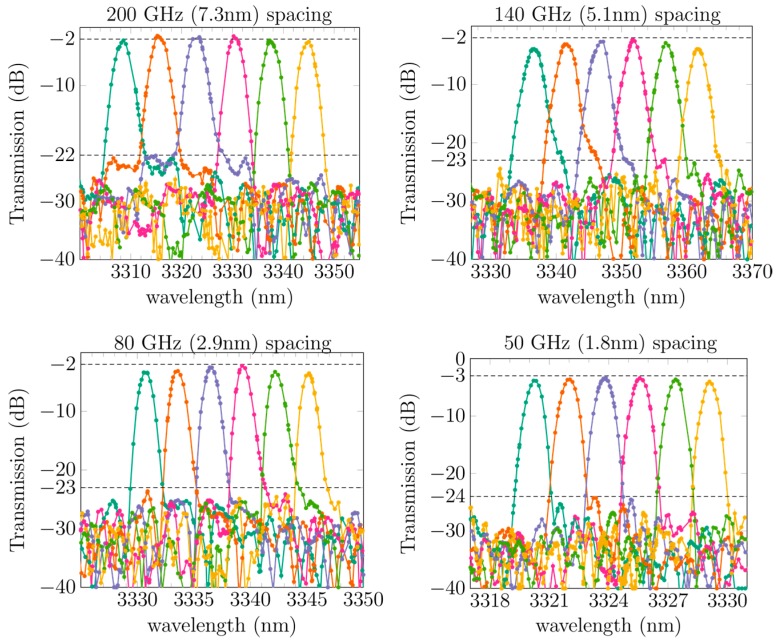
Transmission of four different SOI AWGs operating in the 3.3 μm wavelength range with different channel spacing. The insertion loss (2–3 dB) and crosstalk levels (20–21 dB) are indicated by the dashed lines. The high-resolution (50 GHz) AWG can be used as a mid-infrared DFB laser array multiplexer.

Recently, compact AWG spectrometers in the SOI platform operating in the 2–4 μm wavelength range were demonstrated [63,73,77]. A microscope picture of an SOI AWG operating in the 2.3 μm wavelength range is shown in Figure 12a. The footprint of the device is 0.45 mm^2^. Figure 12b–d show the measured transmission spectra of three AWG spectrometers operating at different wavelengths. All of the devices are realized in the 400 nm silicon waveguide platform. The silicon waveguide circuits are processed on 200 mm SOI wafers in IMEC’s CMOS pilot line. The waveguide loss is around 0.5 dB/cm in the 2–2.5 μm wavelength range and increases to 2.6 dB/cm at 3.3 μm and 3 dB/cm at 3.8 μm. Additionally, high-resolution AWGs at 3.3 μm are demonstrated in Figure 13 [77]. These can potentially be used as DFB laser array multiplexers. Low insertion loss (2 to 3 dB) and low crosstalk (−30 to −20 dB) are obtained in all of the AWG spectrometers. This state-of-the-art performance of the AWG spectrometers indicates that the SOI waveguide platform is ideal for 2–4 μm wavelength range photonic components and integrated circuits. 

**Figure 14:**
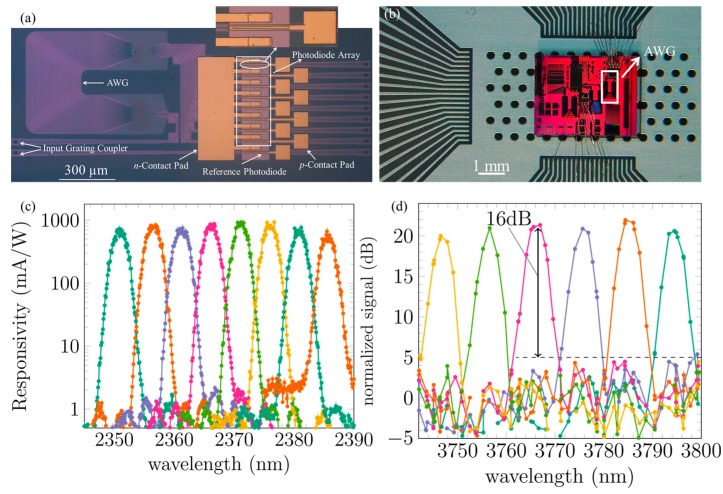
(**a**) Microscope image of the 2.3 μm AWG spectrometer integrated with a InP-based type-II quantum well photodetector array; (**b**) wire bonded III–V-on-silicon AWG spectrometers on a PCB; (**c**) photo-response of the 2.3 μm AWG and (**d**) 3.8 μm III–V-on-silicon AWG spectrometer.

For a spectroscopic sensor, the passive spectrometer should be integrated with photodetectors to convert the optical signals to an electrical response. In addition, the photodetectors should connect with electronic components such as trans-impedance amplifiers to realize a complete opto-electronic system. Figure 14a displays a microscope image of the 2.3 μm AWG spectrometer integrated with an adiabatically-coupled InP-based type-II quantum well photodetector array [63]. Every channel of the AWG is integrated with a photodetector spaced 60 μm apart. In order to interface with the electronic components, the III–V-on-silicon spectrometer is wire bonded to a printed circuit board (PCB), as shown in Figure 14b. A reference photodetector is present on a reference silicon waveguide to estimate the insertion loss of the AWG after heterogeneous integration. The performance of the reference photodetector is identical to the one shown in Figure 8. Figure 14c shows the photo-response of the 2.3 μm III–V-on-silicon spectrometer. During the measurement, the bias voltage is fixed at −0.5 V. An insertion loss of 3 dB and crosstalk level of −27 dB is obtained by normalizing the responsivity to the reference photodetector. This result indicates that the bonding of III–V material on silicon and related post-processes do not degrade the performance of the AWG spectrometer.

To extend the operation wavelength of the III–V-on-silicon AWG spectrometer beyond 3 μm, a heterogeneously integrated InAsSb photodetector was developed [78]. By transferring InAs_0.91_Sb_0.09_ on silicon, a III–V-on-silicon photodetector was realized with a responsivity of 0.3 A/W at 3.8 μm wavelength and a dark current of 170 μA under a bias of −10 mV and 600 μA at −50 mV at room temperature. Figure 14d shows the measured photoresponse of the 3.8 μm AWG spectrometer integrated with an InAsSb photodetector array. Due to the relatively large dark current, optical measurements with a CW laser do not reveal the AWG response clearly. To characterize the device, the light was mechanically chopped and the electrical response was detected with a lock-in amplifier. A crosstalk of −16 dB was achieved. It is believed that the dark current can be reduced by adding barriers in the epitaxial layer stack and by further optimizing the detector passivation.

## 6. On-Chip Mid-Infrared Photothermal Spectroscopy

Over the past two decades, major advances in QCL and ICL technologies have opened the door for highly demanding TDLAS applications such as trace gas detection at ppb levels in the mid-infrared. The longer wavelength range promises better sensitivities and label-free selectivity due to the unique and strong ro-vibrational resonances of each molecule at these frequencies. Miniaturization and improvements of these sources is advancing rapidly [79]. However, sensitive detectors in the infrared remain bulky, expensive and require cooling. One approach for further development of portable and cheap systems is to move away from traditional TDLAS methods to others such as photoacoustic and photo-thermal spectroscopy wherein the ro-vibrational resonance is excited optically and transduced to a local change in pressure or temperature of the analyte. One of the main benefits is that the photo-thermal signal is exactly zero when there is no optical absorption as opposed to traditional transmission spectroscopy, where a small change in transmission needs to be detected. The latter is also heavily affected by scattering and reflection losses which makes it less suitable for applications in the field. Many different realizations of photoacoustic and photo-thermal systems have been shown over the past decade which are extremely sensitive [19,20,21,22,23,24,25,80]. Integrating such systems on a chip is therefore extremely valuable. In this section, a photo-thermal spectroscopy method is discussed which utilizes the mature SOI technology in the telecommunication wavelength range to make a photo-thermal transducer suitable for mid-infrared trace gas spectroscopy applications. A proof of principle measurement was conducted on a polymer analyte in the 3.2–3.6 μm wavelength range to good agreement with traditional Fourier-transform infrared spectroscopy (FTIR) measurements.

**Figure 15:**
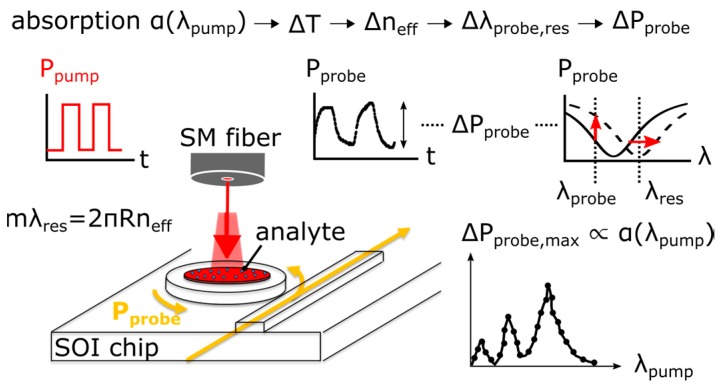
Schematic of the photo-thermal sensing principle. A modulated mid-infrared pump beam is absorbed by the analyte which causes a local temperature change of the microring waveguide. The thermo-optic effect changes the effective index of the waveguide mode, hereby changing the resonance wavelength λ_res_ of the microring. For a given fixed probe wavelength λ_probe_, the change in λ_res_ produces a change in probe power ΔP_probe_ which is measured using a near-infrared detector. The absorption spectrum of the analyte can be reconstructed by scanning the pump wavelength and recording the maximum probe modulation ΔP_probe,max_.

The photo-thermal method is schematically shown in Figure 15. A SOI microring resonator operating at 1.55 μm acts in combination with the analyte as a bolometer. The analyte is placed in the annular region of the MRR which is thermally connected to the ring waveguide through the silicon device layer. A tunable mid-infrared pump laser beam at 3 μm wavelength is chopped and flood illuminates the analyte. The optical absorption gives rise to a temperature change which shifts the resonance wavelength of the MRR. By fixing the probe wavelength on the slope of the MRR transmission, the shift in resonance wavelength is transduced to a power change on the near-infrared probe detector. This power change is proportional to the absorption coefficient of the analyte. The mid-infrared absorption spectrum is recovered by scanning the mid-infrared pump beam while recording the probe modulation amplitude.

The photo-thermal signal scales with the pump and probe powers, optical Q-factor of the MRR and the effective thermal resistance of the MRR waveguide [18,81].

**Figure 16:**
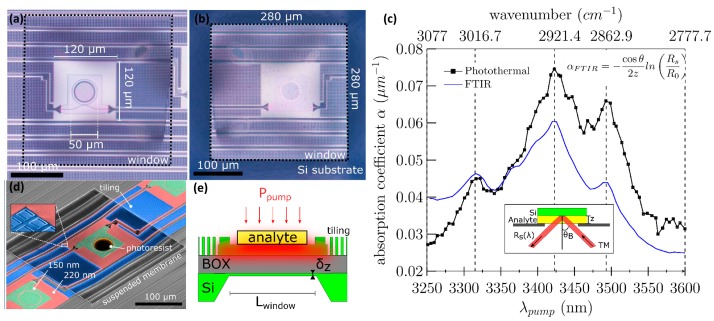
Microscope image of the SOI MRR suspended on BOX membrane with AZ5214 photoresist as mock-up analyte: (**a**) top and (**b**) bottom view; (**c**) The measured photo-thermal signal is scaled to calculate the absorption coefficient of the analyte and is compared to FTIR measurement. The FTIR signal is collected in reflection at the Brewster angle and TM polarization and is used with the formula in the inset to estimate the absorption coefficient; (**d**) A tilted SEM image with false coloring shows the various regions of the PIC; (**e**) A schematic cross section of the suspended MRR is given.

As an initial experiment, a 1.35 μm thick photoresist AZ5214 was lithographically patterned inside the MRR ring area as a mock-up analyte. A MRR with a Q-factor of ~100 k was fabricated and the backside silicon substrate was locally etched in KOH to improve the thermal isolation by a factor of ~40 to approximately 10^4^ K/W, see also Figure 16 [80]. The recovered photo-thermal spectrum was obtained by scanning the wavelength of an optical parametric oscillator (OPO) system operating in the 3 μm wavelength range, but the source could be replaced by a faster and cheaper QCL/ICL operating in a relevant wavelength range. At each mid-infrared wavelength, the pump beam is mechanically chopped and the resulting maximal photo-thermal probe signal is recorded. The pump power exiting the fiber in this experiment was between 0.1 and 1 mW. The signal is scaled with the setup parameters to estimate the absorption coefficient of the analyte to good agreement with a benchmark FTIR measurement, see also Figure 16c. From the measurements, a normalized noise equivalent absorption coefficient NNEA of 7.6 × 10^−6^ cm^−1^ W/Hz^1/2^ was derived.

For real applications, the photoresist could be replaced by a gas-adsorbing porous coating that would capture trace gas amounts [5]. These coatings can have large pre-concentration factors that would boost the Limit of Detection (LOD) for this type of transducers to competitive values. With pre-concentration factors equal to one, these type of transducers are estimated to already have LOD values of a few ppm for trace gases in the 3–4 μm wavelength range.

**Figure 17:**
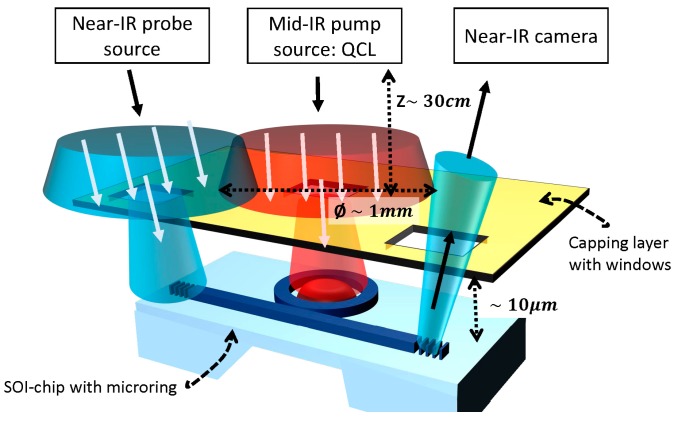
Schematic of a possible free-space measurement configuration. The probe and pump sources flood-illuminate the chip from a certain distance, e.g., 30 cm. The SOI-chip is capped with a reflective or absorbing second layer (e.g., gold-coated silicon) with spacers (not shown in schematic). Small apertures (uncoated areas of the capping layer) are aligned on top of the MRR and the input/output ports of the probe. The probe signal is collected by a near-IR camera.

The proposed transducer could be used as a cheap non-contact all-optical interrogator which is desired in some applications such as trace gas analysis of food packages for early spoilage detection. Furthermore, collimated probe and pump beams in free space can be used, as opposed to fibers, to flood illuminate the transducer from a small distance, see also Figure 17. The optical power budget can be traded for more tolerance on the beam alignment. The diminished optical power could be compensated by the coating pre-concentration factor. The probe signal is collected by a near-IR camera. Alternatively, for a fully integrated sensor solution, the near-infrared laser/photodetector and mid-infrared pump source can be integrated as well, similar to the devices described in Section 4.

## 7. Conclusions

We have reviewed our recent results on mid-infrared silicon photonic integrated circuits for spectroscopic sensing applications in the 2 to 4 μm wavelength range. Low-loss and ultra-compact waveguide circuits can be realized for this wavelength range using the well-established silicon photonics platform. Fully integrated 2.3-μm-wavelength photonic circuits consisting of silicon waveguides, DFB lasers, photodetectors and AWG spectrometers are achieved by integrating InP-based type-II epitaxial layer stack on silicon. In CW regime, the 2.3 μm range III–V-on-silicon DFB operates up to 25 °C and shows an output power of 3 mW in a single mode. By varying the silicon grating pitch, a DFB array with broad wavelength coverage from 2.28 μm to 2.43 μm is realized. Besides, a continuous current-tuning range of more than 10 nm can be achieved by fabricating four DFB lasers with different waveguide widths. Heterogeneously integrated photodetectors with the same epitaxial layer stack exhibit a responsivity of 1.6 A/W near 2.35 μm and dark current of 10 nA at −0.5 V. Besides heterogeneous integration, butt-coupling a GaSb-based gain chip with a silicon photonic IC also provides a solution to realize a compact 2 μm range silicon photonic light source. In this way, a GaSb/silicon external cavity laser with a 58 nm tunable range and SMSR better than 52 dB over the whole range is realized. The high-index-contrast SOI platform also enables ultra-compact 2–4 μm wavelength range AWG spectrometers. Low insertion loss (2–3 dB) and low-crosstalk (20–30 dB) AWG spectrometers were demonstrated for 2–4 μm wavelength range. Integrating these AWGs with III–V photodetectors does not degrade the performance of the spectrometers. 

A novel photothermal spectroscopic transducer is discussed. By using a high-Q factor SOI MRR operating at 1.55 μm which probes the heat generated through optical absorption, we circumvent the need of using a cooled mid-infrared detector for spectroscopy applications at longer wavelengths. Initial results show good agreement with benchmark FTIR measurements in the 3–4 μm wavelength range. By suspending the MRR on the BOX layer, the thermal isolation of the transducer is increased and a NNEA of 7.6 × 10^−6^ cm^−1^ W/Hz^1/2^ is estimated. Integration of this type of transducer with gas adsorbing porous coatings is a promising approach for sub-ppm trace gas detection. For a comprehensive overview on different spectroscopic sensing measurements we refer the reader to [13].
